# A novel tamoxifen-inducible Mct8-CreERT2 mouse model for targeted studies of Mct8-expressing cells and thyroid hormone transport and function

**DOI:** 10.1007/s11248-025-00471-8

**Published:** 2025-11-28

**Authors:** Anna Molenaar, Noémi Mallet, Marin Bralo, Luciano J. Hoeher, Sonja C. Schriever, Ekta Pathak, Miriam Bernecker, Timo D. Müller, Ali Ertürk, Alberto Cebrian-Serrano, Paul T. Pfluger

**Affiliations:** 1 Research Unit NeuroBiology of Diabetes, Helmholtz Munich, Neuherberg, Germany; 2 Institute for Diabetes and Obesity, Helmholtz Munich, Neuherberg, Germany; 3 German Center for Diabetes Research (DZD), Neuherberg, Germany; 4 Division of NeuroBiology of Diabetes, TUM School of Medicine and Health, Technical University Munich, Munich, Germany; 5 Institute for Intelligent Biotechnologies (iBIO), Helmholtz Munich, Neuherberg, Germany; 6 Institute for Stroke and Dementia Research, Klinikum der Universität München, Ludwig-Maximilians University Munich, Munich, Germany; 7 Munich Cluster for Systems Neurology (SyNergy), Munich, Germany; 8 Walther-Straub-Institute for Pharmacology and Toxicology, Ludwig-Maximilians-University Munich, Munich, Germany; 9 Deep Piction, Munich, Germany; 10 School of Medicine, Koç University, Istanbul, Turkey

**Keywords:** MCT8, Thyroid hormone, Choroid plexus, Tanycyte, Cre

## Abstract

Deficiency of the Monocarboxylate Transporter 8 (MCT8) severely impairs thyroid hormone (TH) transport into the brain, disrupting brain development as well as peripheral TH homeostasis. Studies assessing MCT8 expression patterns and tissue-specific pathologies induced by local TH-deficiency are often inconclusive due to unreliable antibody staining and the lack of functional tools to specifically target MCT8-expressing cells. For this purpose, we generated non-inducible Mct8-Cre and tamoxifen-inducible Mct8-CreERT2 mice. Mct8-Cre;Sun1-sfGFP mice demonstrated ubiquitous Sun1-sfGFP expression, due to early recombination driven by Mct8 gene expression at the stage of trophoblast implantation. Tamoxifen injection in 6-week-old Mct8-CreERT2 mice induced reporter expression specifically in Mct8-expressing cells in the brain and peripherally in liver, kidney, and thyroid, without leaky reporter expression in vehicle controls. Using vDISCO tissue clearing and 3D-imaging of GFP-nanobody-boosted mice, we further identified the sublingual salivary gland and the prostate as prominent Mct8-expressing organs. Nuclei from Mct8-expressing cells in the brain could selectively be enriched using fluorescence-activated nuclei sorting on Mct8-CreERT2;Sun1-sfGFP mice and characterized as choroid plexus cells and tanycytes. Our new inducible Mct8-CreERT2 line provides researchers with a tool to reliably mark, enrich, and characterize Mct8-expressing cells and to genetically modify genes specifically in these cells to study thyroid hormone transport and function.

**Supplementary Information:**

The online version contains supplementary material available at 10.1007/s11248-025-00471-8.

## Introduction

The monocarboxylate transporter 8 (MCT8) is an influx and efflux transmembrane transporter for thyroid hormones (TH) thyroxine (T_4_) and 3,3′,5-triiodothyronine (T_3_), and their metabolites 3,3′,5′-triiodothyronine (rT_3_) and 3,3′-diiodothyronine (Friesema et al. 2003). Its role in TH transport is crucial for brain TH availability, evidenced by the devastating neurological impairments of Allan-Herndon-Dudley-Syndrome (AHDS) patients with mutations in the MCT8-encoding *solute carrier family member 16a2* (*Slc16a2*) gene on the X-chromosome (Dumitrescu et al. 2004; Friesema et al. 2004). Deficient TH transport during crucial stages of brain development causes intellectual disability, motor dysfunctions, and impaired myelination. Reduced TH brain levels are thereby contrasted by elevated T3 plasma levels, causing peripheral thyrotoxicosis (Bernal et al. 2015).

To understand TH transport and its associated diseases, it is important to elucidate the expression pattern of MCT8 in the brain as well as other organs across various developmental stages. Western Blotting found highest levels in the human liver, followed by pituitary and brain, low signals for the heart, and near absence in the lung (Wirth et al. 2009). MCT8-mapping to the human brain, thyroid, and pituitary was moreover confirmed using immunohistochemistry (IHC) and immunofluorescence (IF) (Alkemade et al. 2006; López-Espíndola et al. 2019; T. Wang et al. 2023a, b; Y. Wang et al. 2023a, b; Wirth et al. 2009, 2011).

Murine *Slc16a2* mRNA levels are high in liver and kidney, low in lung, cerebral cortex, and heart, and barely detectable in testis (Mouse ENCODE transcriptome project PRJNA66167) (Yue et al. 2014). Mct8 protein was localized in rodent livers, thyroid glands, kidneys, brains, and to a lesser extent rat hearts (Di Cosmo et al. 2010; Friesema et al. 2003; Wilpert et al. 2020; Wirth et al. 2011) (Supplemental Table 1). IHC and IF in adult human brains revealed abundant MCT8 levels in barrier cells (endothelial cells, astrocytes, choroid plexus (ChP), tanycytes), clearly marking blood vessels (BV) and capillaries, but sparse neuronal staining (López-Espíndola et al. 2019; T. Wang et al. 2023a, b; Y. Wang et al. 2023a, b). Neuronal MCT8 signal was readily detectable in the fetal brain (Wirth et al. 2009) and in human cortical organoids (Graffunder et al. 2024).

In murine brains, Mct8 protein was abundant in BV, tanycytes, and ChP, while neuronal expression declined from postnatal day 12 (P12) to P21, until undetectable (Wilpert et al. 2020). Others found Mct8 in the adult mouse hippocampus and Purkinje cells and, diffusely, in cortical neurons (Wirth et al. 2009). Discrepancies in Mct8 detection are likely due to differences in antibodies and lots used, highlighting the need for alternative visualization methods due to challenges in acquiring effective antibodies and inconsistent neuronal staining (Wilpert et al. 2020). We here explored the use of the Cre-lox system, where expression of Cre recombinase is driven by the endogenous *Slc16a2* promoter, and crossed these with fluorescent reporter mice to identify, visualize and characterize *Slc16a2*-expressing cells in vDISCO tissue cleared and 3D imaged whole mice and isolated organs.

## Results

### Generation of tamoxifen-inducible Mct8-CreERT2 mice

**Fig. 1:**
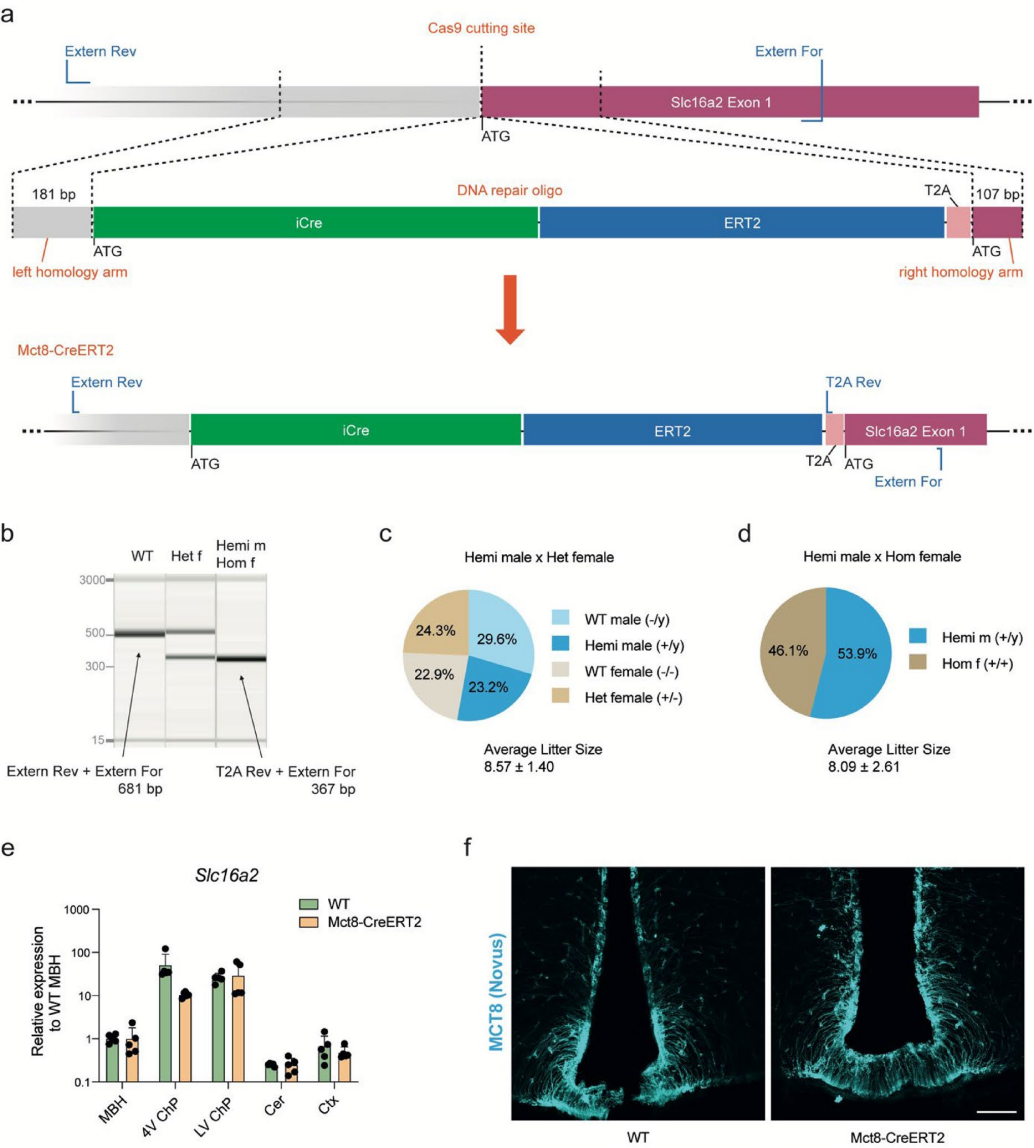
Generation and characterization of transgenic Mct8-CreERT2 knock-in mice. **a** CRISPR-Cas9 guided insertion of *iCreERT2* and *T2A* sequences at the start codon of the Mct8 gene *Slc16a2*. **b** Representative gel of genotyping results using primers (see Supplemental Table 2) to discriminate the hemizygous (Hemi, +/y) male or homozygous (Hom, +/+) female mice from heterozygous (Het, +/−) females and from the WT mice, using primers indicated in **a** in blue. “+” indicates the mutant allele, “−” the WT allele on the X-chromosome. **c** Ratios of offspring from the mating of Hemi males with Het females (left) or **d** Hemi males with Hom females (right). Litters of 7 females for each type of mating were assessed, with a total of 60 and 89 pups, respectively. **e** Relative gene expression levels of *Slc16a2* in the mediobasal hypothalamus (MBH), ChP of the 4th ventricle (4 V ChP) and lateral ventricle (LV ChP), cerebellum (Cer) and cortex (Ctx) of WT and Mct8-CreERT2 mice, quantified using qPCR and the ddCT method relative to *Malat1*. **f** Immunofluorescent staining for Mct8 using the Novus antibody in hypothalamic slices of WT and Mct8-CreERT2 mice. **e** Statistical analysis using multiple t-tests with Holm-Sidak correction, N = 5 per genotype, showing no significant changes at *p* < 0.05. **f** Scalebar = 100 µm

Prompted by discrepant studies on the presence or absence of neural Mct8 protein expression in adult mice (Supplemental Table 1) and our own comparison of commercially available antibodies, which revealed unspecific or insufficient immunofluorescence signals for Mct8 in murine brain slices (Supplemental Fig 1), we aimed to generate a novel Mct8-CreERT2 mouse line to ultimately assess Mct8 expression patterns in adult mice. Mct8-CreERT2 mice were generated by inserting the sequences of *iCreERT2* and “self-cleaving” peptide *T2A* at the start codon of Mct8-gene *Slc16a2* using CRISPR/Cas9 (Fig. 1a) (Donnelly et al. 2001; Hayashi and McMahon 2002; Jahn et al. 2018; Shimshek et al. 2002; Szymczak et al. 2004), generating viable mice at the expected Mendelian ratio and with the correct genotypes (Fig. 1b–d). The transgenic mice had comparable levels of *Slc16a2* mRNA in WT male and Mct8-CreERT2^+/y^ brain areas (Fig. 1e, Supplemental Fig. 2a), with highest levels in the ChP of the 4th ventricle (4 V ChP) and lateral ventricle (LV ChP), followed by the mediobasal hypothalamus (MBH), then cortex (Ctx) and cerebellum (Cer), consistent with published Mct8 gene expression levels (Müller and Heuer 2014). Likewise, we observed unperturbed endogenous Mct8 protein expression, with comparable fluorescence intensities in antibody-stained hypothalamic slices of WT and Mct8-CreERT2 mice (Fig. 1f). The localization of Mct8 at the cell body and along the processes of tanycytes is conserved in the knock-in mice. Similarly, the expression of Mct8 at the apical membrane of the ChP is undisturbed (Supplemental Fig. 2b).

### Ubiquitous recombination in the CNS of non-inducible Mct8-Cre mice

Using the same CRISPR-Cas9-based knock-in strategy, we explored germline *Slc16a2* expression patterns using non-inducible Mct8-Cre mice. In the fully viable and fertile Mct8-Cre mice crossed with the fluorescent reporter mouse line Sun1-sfGFP, which expresses the GFP-tagged nuclear lamina protein Sun1, we found ubiquitous reporter expression in adult mouse brains (Supplemental Fig. 3). We next investigated whether this ubiquitous pattern could be due to a very early activation of Mct8-Cre expression and subsequent stop cassette excision in all cells of that lineage. Blastocysts (corresponding to embryonic day E4.5) from the mating of Mct8-Cre with Ai14 mice, known for their strong cytosolic fluorescence, showed no reporter signal, while Mct8-Cre;Sun1-sfGFP embryos at stages E10.5 had reporter expression in virtually all cells (Supplemental Fig. 4a, b). By analyzing publicly available RNA sequencing data across murine embryonal developmental stages, we identified a peak for the expression of *Slc16a2* at day E5.25, i.e. the stage of implantation directly after the blastocyst stage, to likely drive fluorescent reporter activation (Supplemental Fig. 4c).

### Validation of CreERT2 recombinase activity in *Slc16a2*-expressing cells in the brain

**Fig. 2:**
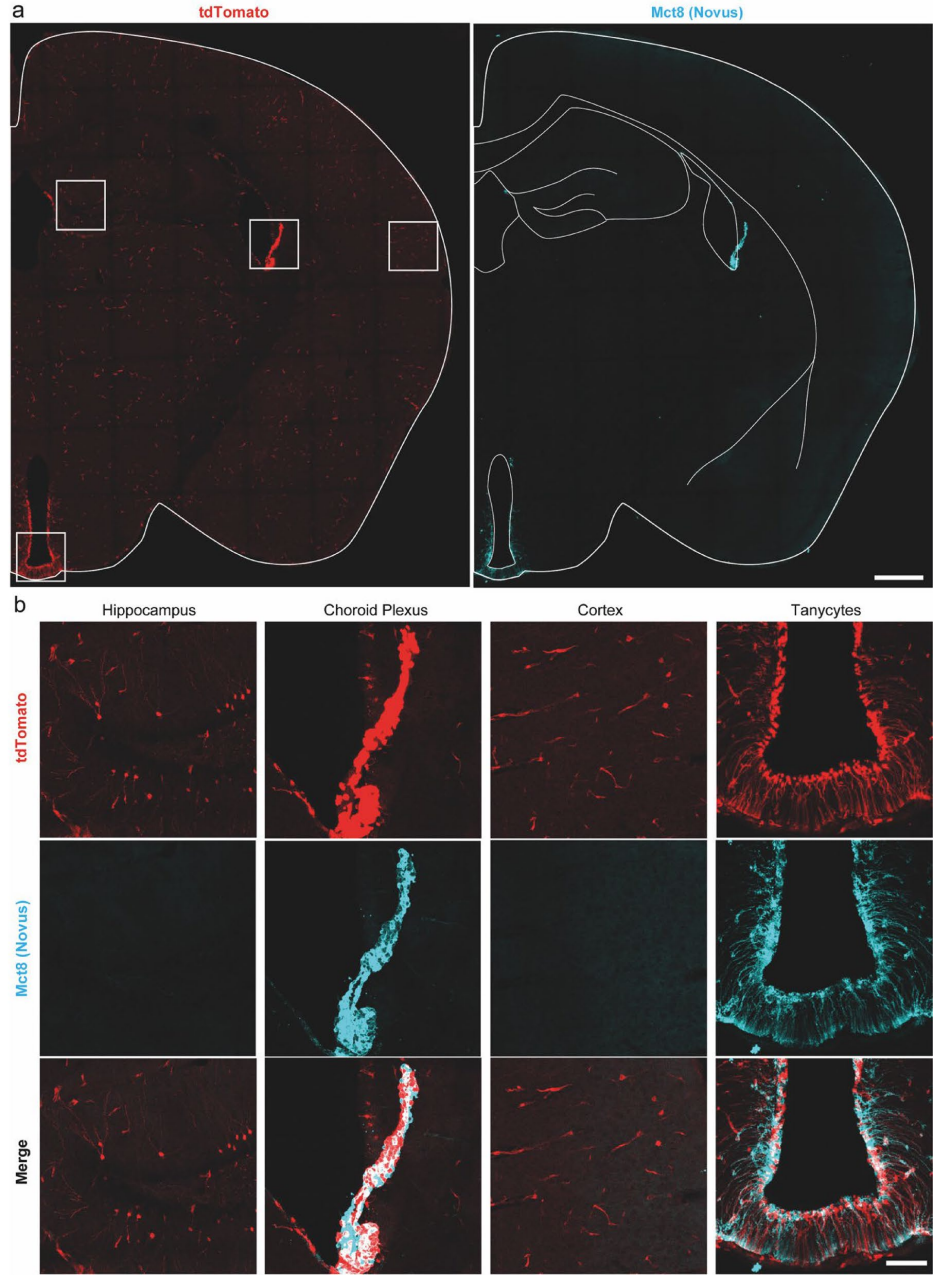
Mct8-CreERT2 driven tdTomato signal in adult mouse brains. **a** Comparison of the tdTomato pattern (left) in a brain slice of an Mct8-CreERT2;Ai14 mouse induced with 1 mg TAM i.p. for three days with Mct8 antibody staining (right; Novus). Areas magnified in **b** are marked with boxes (left); lines (right) included for anatomical guidance. **b** Magnifications of the reporter expression (top), the Mct8 antibody staining (middle) and merge of both (bottom) for the hippocampus, ChP, cortex, and tanycytes. **a** Scalebar = 500 µm. **b** Scalebar = 100 µm

To validate the Mct8-CreERT2 mouse line’s specificity, we crossed it with the fluorescent Ai14 and Sun1-sfGFP reporter mouse lines and assessed CreERT2 activity one week after the application of tamoxifen (TAM) by a single oral gavage (o.g.), a single intraperitoneal (i.p.) injection, or three consecutive i.p. injections of 1 mg TAM per mouse, respectively. In brains of 3 × TAM-injected Mct8-CreERT2;Ai14 and Mct8-CreERT2;Sun1-sfGFP mice, we found profound fluorescence reporter expression in tanycytes and ChP that largely overlapped with Mct8 antibody staining (Fig. 2a, b; Supplemental Figs. 5a–c, 6). Fluorescence was moreover found in BV and few neurons spattered across the hippocampus, cortex, and striatum. Reporter signal could also be found in the upper part of the subventricular zone (SVZ) (Supplemental Fig. 2c, d). Neither BV nor sparse neurons were labeled using antibody staining. Vehicle (VEH)-injected Mct8-CreERT2^+^ and Mct8-CreERT2^−^ mice that received TAM showed minimal Cre-activity, confirming the highly specific, inducible expression without background activity for both reporter lines.

Last, we found comparable recombination efficacies in response to a single i.p. or o.g. application of TAM. However, reporter expression was profoundly diminished compared to the mice injected with three i.p. doses of TAM (Supplemental Fig. 5a, b). Overall, these data show that the new Mct8-CreERT2 line is functional and faithfully induces reporter expression in cells known to express *Slc16a2* in adult mice after three i.p. doses of 1 mg TAM per mouse.

### Mct8-CreERT2-driven reporter expression in whole mice

**Fig. 3:**
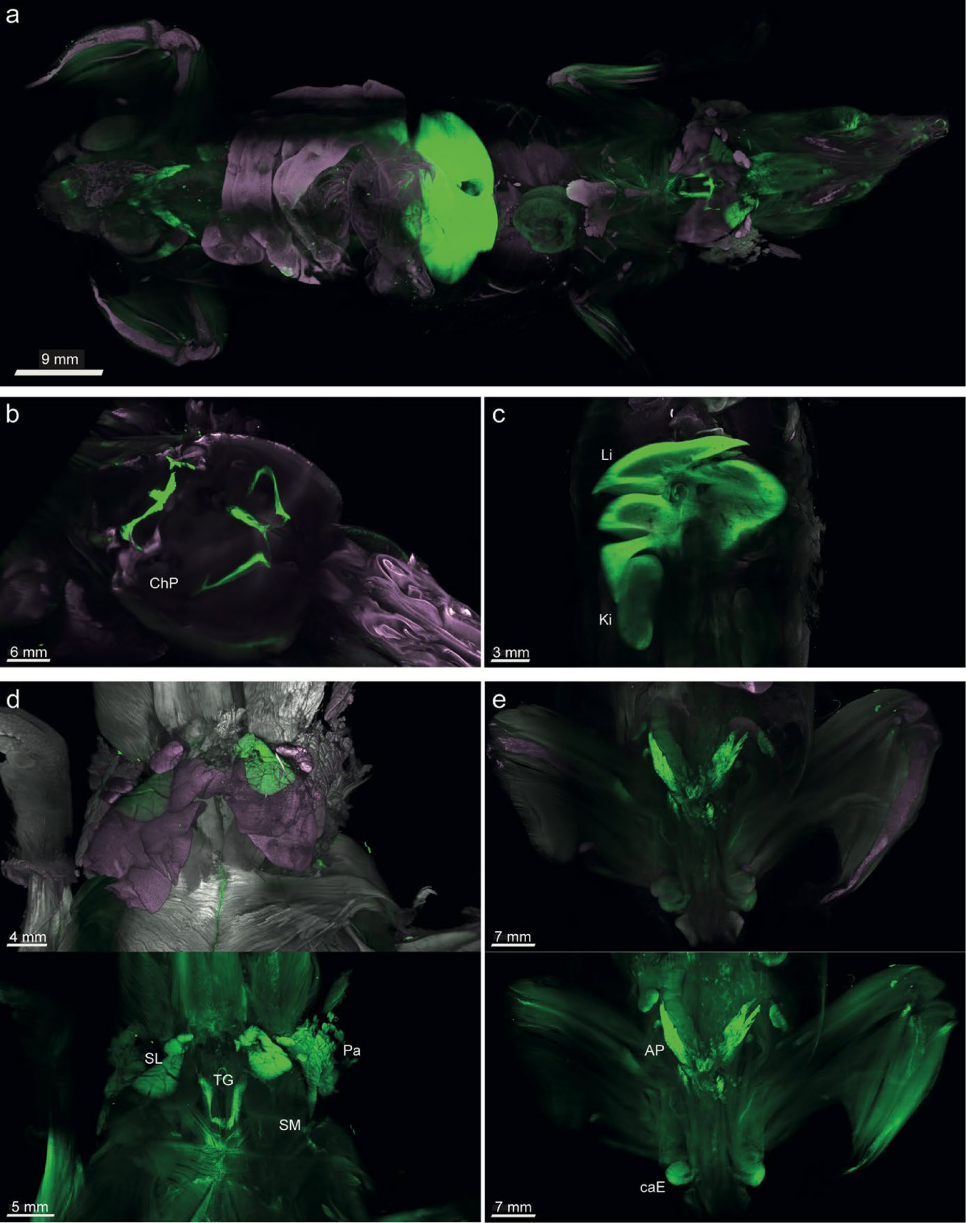
Whole body imaging of TAM-induced Mct8-CreERT2;Sun1-sfGFP mouse using vDISCO and nanobodies directed against GFP. **a** Light Sheet Fluorescence Microscopy (LSFM) of an entire MCT8-CreERT2;Sun1-sfGFP mouse injected three times with 1 mg TAM i.p. at 4 × magnification. LSFM of the **b** head region, showing strong fluorescence in the ventricular ChP, and **c** in a section through liver and kidney. **d** LSFM of the neck region showing strong fluorescence in the sublingual and parotid but not submandibular salivary gland, and in the thyroid gland. **e** LSFM of the lower body showing strong fluorescence in the anterior prostate and cauda epididymis. ChP: choroid plexus, Li: liver, Ki: kidney, SL: sublingual salivary gland, TG: thyroid gland, Pa: parotid gland, SM: submandibular salivary gland, AP: anterior prostate, caE: cauda epididymis. Purple: propidium iodide (PI), white: autofluorescence. (a-c) combination of all three channels (nanobody-enhanced Sun1-sfGFP, PI, autofluorescence), (d,e) top: combination of all three channels, bottom: channel for nanobody-enhanced Sun1-sfGFP reporter detection

After validating the correct reporter expression in mouse brains, we aimed to systematically assess all other *Slc16a2*-expressing tissues. We subjected Mct8-CreERT2;Sun1-sfGFP mice injected thrice i.p. with TAM to vDISCO, the solvent-clearing of organs and nanobody-based immune staining of GFP-marked nuclei, followed by light sheet fluorescent microscopy (LSFM). The whole-body scan is visualized in Fig. 3a, videos of the head, torso and whole-body are provided as Supplemental Material (Supplemental Videos 1–3). Strongest fluorescent signals could be localized to the ChP (Fig. 3b), liver and kidney (Fig. 3c). Figure 3d (upper panel) depicts the distinctly labeled sublingual and parotid salivary glands and the thyroid gland. We further observed strong fluorescence in the prostate, as well as the cauda of the epididymis (Fig. 3e)*.*

**Fig. 4:**
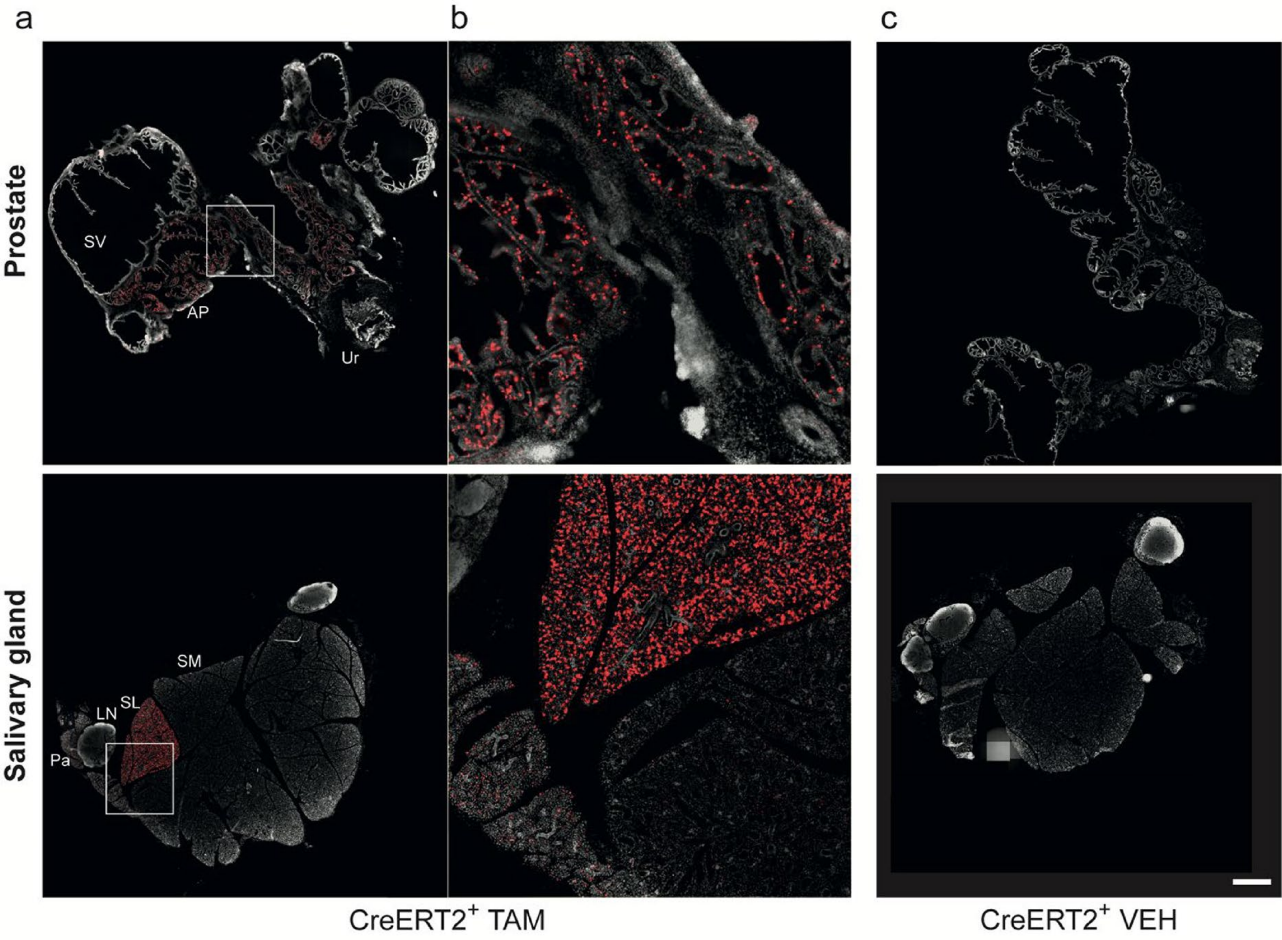
Strong fluorescent reporter expression in prostate and salivary glands of Mct8-CreERT2;Ai14 mice. **a** Cryo-slices of tissues from Mct8-CreERT2;Ai14 mice injected for five days with 1 mg TAM i.p. were assessed for fluorescence signals in the prostate (top) including seminal vesicles, and the salivary glands (bottom). **b** Zoom on areas marked with white square in **a**. **c** Cryo-slices of Mct8-CreERT2;Ai14 mice injected for five days with vehicle (VEH). SV: seminal vesicles, AP: anterior prostate, Ur: urethra, Pa: parotid gland, LN: lymph node, SL: sublingual salivary gland, SM: submandibular salivary gland. Grey: DAPI, red: tdTomato. Scalebar = 1 mm. Exposure time of 555 laser: 0.7 ms

Intrigued by the detection of reporter signal in the salivary glands and prostate via vDISCO, we examined those tissues in more detail using Mct8-CreERT2; Ai14 mice and fluorescence imaging of cryo-slices. We also included organs that were reported to have *Slc16a2* transcripts but showed no or weak reporter expression in the whole mouse scan, namely heart, lung, testis, and skeletal muscle. Of note, we also assessed whether five doses of TAM could augment Cre-activity to maximize reporter expression compared to the 3 × TAM-treated mice but found no differences in fluorescence intensities (Supplemental Fig. 5c). In the prostate, we found strong fluorescence in cells of the anterior prostate and weak fluorescence in seminal vesicles (Fig. 4 top). In the salivary glands, reporter expression was strongest in the sublingual, modest in the parotid, and near-absent in the submandibular gland (Fig. 4 bottom).

**Fig. 5:**
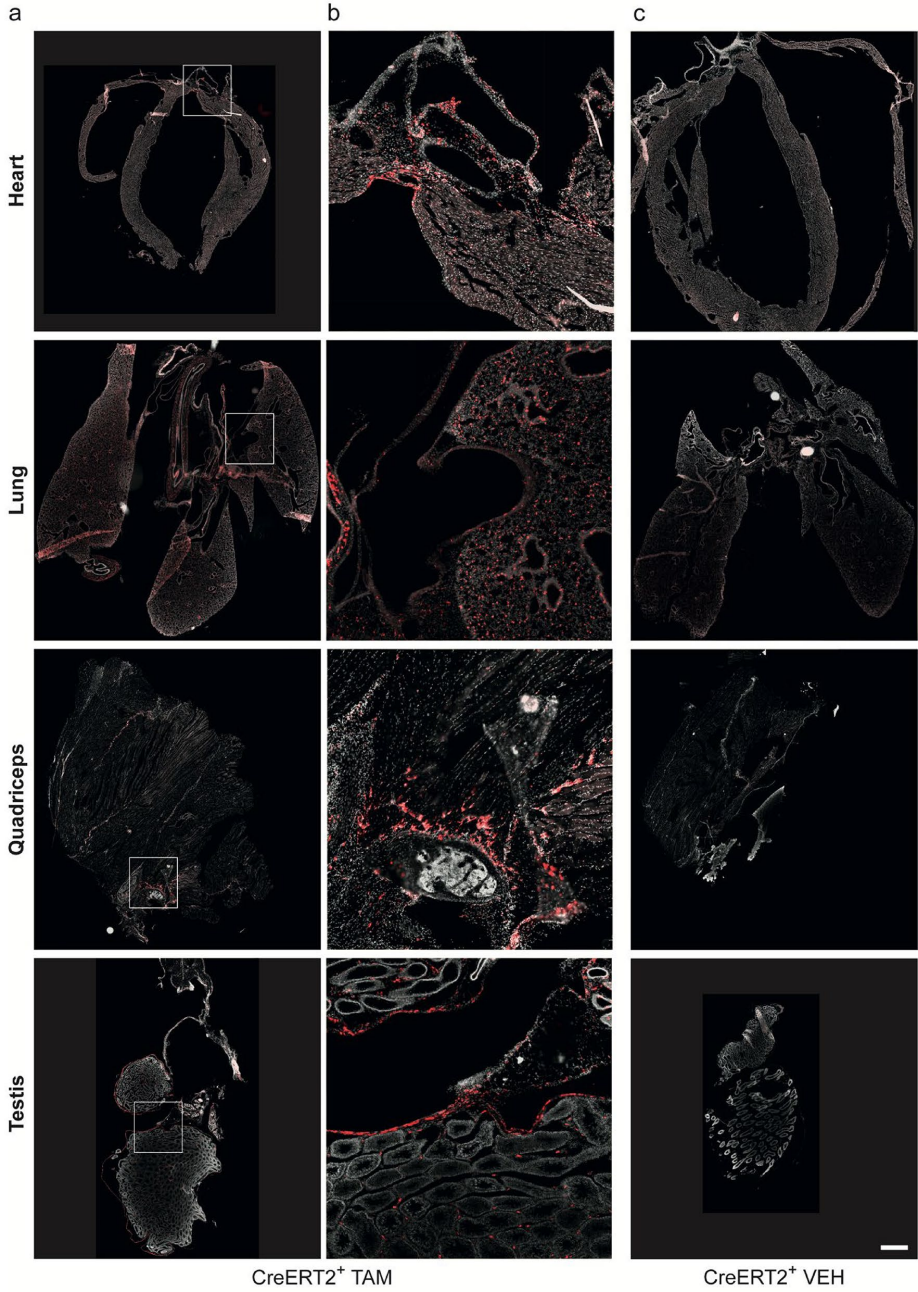
Fluorescent reporter expression in *Slc16a2*-expressing organs of Mct8-CreERT2;Ai14 mice. **a** Cryo-slices of organs from Mct8-CreERT2;Ai14 mice injected five times with 1 mg TAM i.p., from top to bottom: heart, lung, quadriceps including femur (Fe), testis (Te) including caput epididymis (ctE). **b** Zoom on areas marked with white square in **a**. **c** Cryo-slices of Mct8-CreERT2;Ai14 mice injected for five days with VEH. Scalebar = 1 mm. Exposure time of 555 laser: 20 ms

Heart, lung, testis, and quadriceps demonstrated sporadic reporter activation, including signals from the femur and caput epididymis (Fig. 5). However, exposure times had to be approximately 30 × higher than those necessary for the salivary gland and prostate, indicating a sparsely distributed and comparably lower *Slc16a2* expression in those tissues.

### Enriching nuclei of *Slc16a2*-expressing cells from Mct8-CreERT2;Sun1-sfGFP mice using fluorescence-activated nuclei sorting

**Fig. 6:**
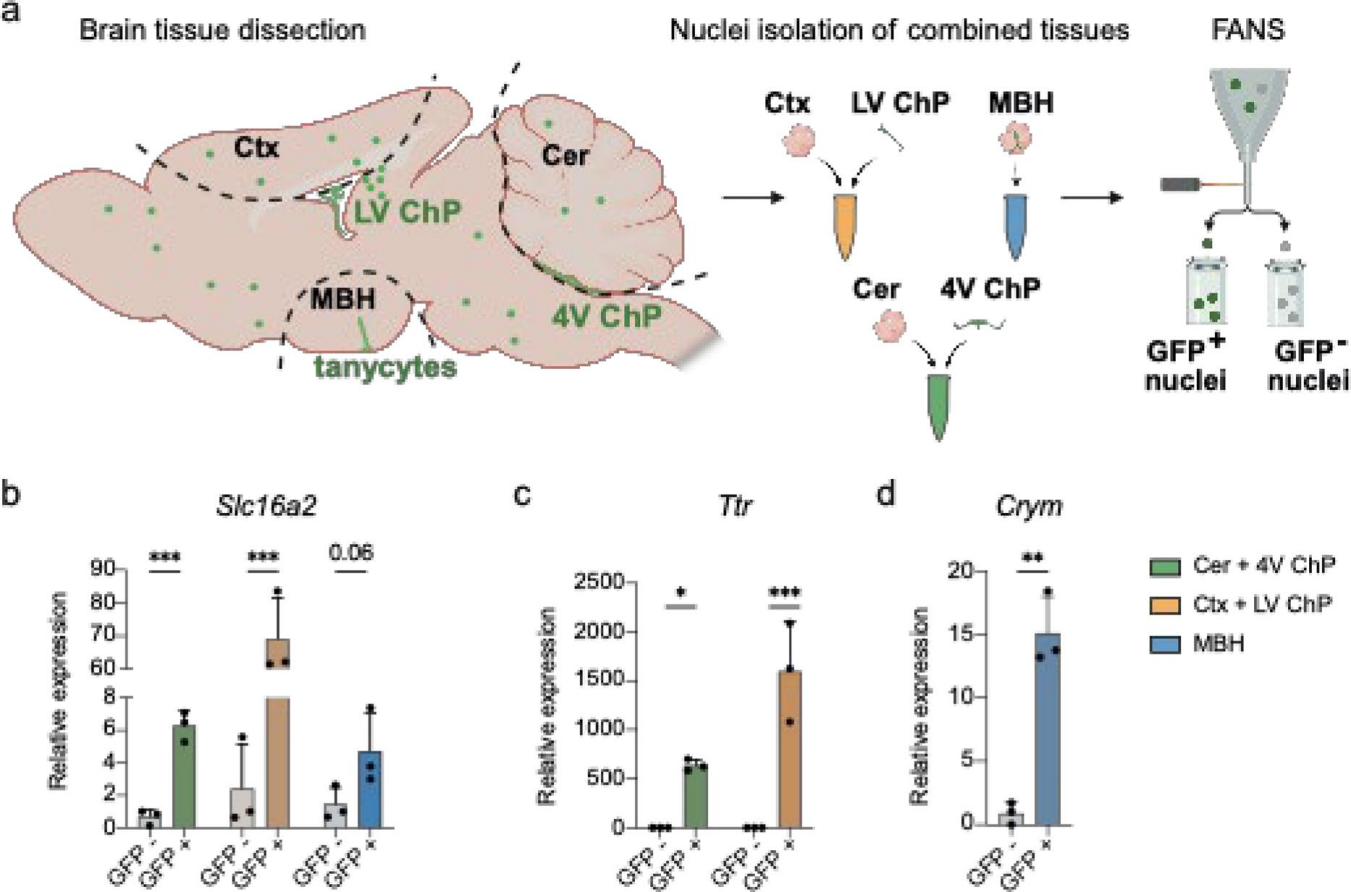
Fluorescence-activated nuclei sorting (FANS) of GFP^+^ nuclei from brain areas of induced Mct8-CreERT2;Sun1-sfGFP mice. **a** Scheme of tissue dissection. The whole Cer and MBH and part of the Ctx were extracted along the dashed lines. Before Cer and Ctx were taken, the 4 V ChP and LV ChP were isolated with tweezers and added to the Cer and Ctx collection tubes, respectively. The samples were then subjected to FANS to collect 1000 nuclei per GFP^+^ and GFP^−^ fraction, respectively. **b** qPCR for *Slc16a2* mRNA levels in GFP^+^ and GFP^−^ fractions of the MBH, Ctx + LV ChP, and Cer + 4 V ChP. (c,d) ChP marker transthyretin (*Ttr*) mRNA levels in the Ctx + LV ChP and Cer + 4 V ChP samples. **d** Tanycyte marker µ-crystallin (*Crym*) mRNA levels in the MBH. qPCR analyses were done using the ddCT method relative to *Malat1*, relative to a GFP^−^ reference sample per tissue type. Statistical analyses were done using multiple t-tests with Holm-Sidak correction. *p < 0.05, **p < 0.01, ***p < 0.001. N = 3 per genotype

Finally, we assessed whether the line can be utilized for the fluorescence-activated nuclei sorting (FANS)-based enrichment of *Slc16a2*-expressing cells from selected brain areas of TAM-induced Mct8-CreERT2;Sun1-sfGFP mice (3 × 1 mg, i.p.). *Slc16a2*-positive nuclei, stably tagged with Sun1-sfGFP anchored in the nuclear envelop, were collected from the mediobasal hypothalamus (MBH) that includes multiple *Slc16a2*-express ingtanycytes. For the Ctx and the Cer, where stainings showed minimal MCT8 protein levels, we included the MCT8-rich lateral ventricle ChP (LV ChP) and 4th ventricle ChP (4 V ChP), respectively, to ensure sufficient GFP + nuclei for the RNA extraction and qPCR. Per tissue combination and mouse we sorted 1000 GFP + and 1000 GFP- nuclei (Fig. 6a). An exemplary gating for GFP^+^ nuclei is shown in Supplemental Fig. 7. All GFP^+^ fractions showed strong *Slc16a2* mRNA enrichment compared to the GFP^−^ fractions, except for the MBH sample which did not reach significance (Fig. 6b). qPCR for ChP marker transthyretin (*Ttr*) and tanycyte marker µ-crystallin (*Crym*) showed enrichment of ChP- and tanycyte-derived nuclei in the GFP^+^ fractions of the Ctx + LV ChP, Cer + 4 V ChP, and MBH, respectively (Fig. 6c).

## Discussion

Our new TAM-inducible Mct8-CreERT2 mouse line allows specific recombination in all *Slc16a2*-expressing cells throughout the body with high specificity and minimal leakiness. By inserting the iCreERT2 cassette directly into the endogenous *Slc16a2* locus, we preserve physiological Mct8 expression while enabling controlled iCreERT2 activity. This knock-in strategy offers a clear advantage over traditional approaches relying on bacterial artificial chromosomes (BACs) transgenesis or random transgene integration, which frequently cause unpredictable expression patterns, position effects, gene silencing, or disruption of endogenous loci (Gandhi et al. 2024; Halurkar et al. 2025; McLeod et al. 2020). Such artifacts can introduce major confounders and compromise the physiological relevance of experimental models. In contrast, our targeted approach faithfully recapitulates endogenous *Slc16a2* regulation, minimizing off-target effects and preserving the native biology of the system.

The non-inducible Mct8-Cre line showed ubiquitous reporter expression due to the early *Slc16a2* expression peak at day E5.25 of embryonal development. This indicates an important, vaguely understood role of TH transport during nidation when embryonal trophoblast cells invade the mother’s endometrium to form the placenta. Notably, Chan et al. (Chan et al. 2006) found that *Slc16a2* expression is increased in the placentas of humans with intrauterine growth restriction, to potentially increase T3 uptake, and embryos of Mct8-KO rats showed a decreased embryo-to-placental weight ratio. These effects were nonetheless mild, and little evidence pointed toward an impaired trophoblast invasion capability in the KO rats (Vasilopoulou et al. 2013). This may be due to organic anion transporting polypeptide 1c1 (Oatp1c1), a transporter for T3 and T4 co-expressed in the rat placental barrier that can partially compensate for the lack of Mct8 in rats and mice but not in humans (Y. N. Sun et al. 2014). For the human placenta, MCT10 has been reported as a major contributor to TH-transport, and other transporters like LAT1 and LAT2 have also been found to play a role (Chen et al. 2022). Future studies are thus warranted to elucidate the specific role of MCT8 compared to other transporters in trophoblast invasion and placental development in humans.

In the brains of TAM-induced Mct8-CreERT2 fluorescent reporter mice, we found strong and specific fluorescence in tanycytes and ChP one week after induction, confirmed by Mct8 antibody staining. Additionally, we found reporter signal in BV, scarcely in neurons of the hippocampus, cortex, and striatum, fitting to reported cell types expressing *Slc16a2* (Wirth et al. 2009)(Wilpert et al. 2020)(Müller and Heuer 2014), and in the SVZ, which is consistent withreports on Mct8 expression in SVZ neural stem cells (Luongo et al. 2021). We further detected strong reporter signal in liver, thyroid gland, and kidney (Di Cosmo et al. 2010; Friesema et al. 2003; Trajkovic-Arsic et al. 2010). The Encyclopedia of DNA Elements (ENCODE) database reports very low *Slc16a2* transcripts in mouse lung, heart, and testes, compared to strongly expressing tissues (Yue et al. 2014). Similarly, *Slc16a2* expression was reported for various muscle cells using microarrays, but protein staining was only reported for satellite cells (Mayerl et al. 2018). In our Mct8-CreERT2 reporter mice, fluorescent signals in such tissues and organs with low gene expression were weak, but detectable. Our mouse model is thus coherent with physiological expression patterns, and a suitable tool to target both organs with a high number of *Slc16a2*-expressing cells as well as organs with a low abundance of *Slc16a2*-expressing cells.

We also found profound reporter signal in organs where a role for Mct8 was not previously described, the sublingual salivary gland and the prostate. Salivation is influenced by TH both in rodents and in humans (Carbone et al. 1966; Muralidharan et al. 2013; Westmuckett et al. 2013), and TH is found in saliva (Sawicka-Gutaj et al. 2024). Our data are consistent with those reports, and highlight that the sublingual and, to a lesser extent, the parotid salivary glands are involved in that salivary TH transport. The prostate secretes fluids that are added to the seminal fluid to facilitate sperm fertility (Pang et al. 1979). Prostate fluids also contribute to the generation of copulatory plugs, which is essential for the successful fertilization in mice (Cukierski et al. 1991). The role of Mct8 in male reproduction was already studied in Mct8-KO rats, where Mct8-staining was reported for the rat epididymis. That study explored the role of Mct8 in testes and epididymis for sperm viability and reported a decrease in fertility in male KO rats (Bae et al. 2020). Our data highlight that such changes in fertility could also potentially be attributed to the lack of Mct8 in the prostate.

ast, the strong enrichment of *Slc16a2*, and the selective enrichment of ChP and tanycytes markers in the GFP^+^ fraction of spiked Ctx and Cer samples ultimately confirm that our Mct8-CreERT2 line combined with Sun1-sfGFP reporter mice is a useful model to enrich *Slc16a2*-expressing nuclei using FANS. Combined with downstream applications such as single nucleus RNA sequencing, this may one day help to fully elucidate the identity and function of *Slc16a2*-expressing cells in the brain and peripheral organs. Likewise, Mct8-CreERT2 mice can be used to generate conditional gene knock-out or overexpression models specifically in *Slc16a2*-expressing cells, to study their physiological relevance in TH biology in those cells. To conclude, our new mouse model is a valuable genetic tool that can facilitate the study of TH biology and its transport mechanisms, ultimately providing crucial insights into the physiological roles of *Slc16a2*-expressing cells.

## Limitations

This paper describes the initial validation of a MCT8-CreERT2 mouse line. Our initial characterization focused solely on male mice, as pathogenic mutations in MCT8 predominantly cause disease in male patients, with only rare cases reported in females. Accordingly, future studies need to overcome that limitation by also studying *Slc16a2* gene expression patterns in female mice. Another limitation warranting additional studies is the low sample sizes for enrichment of *Slc16a2*-expressing nuclei using FANS. We had to spike the Ctx and Cer with ChP tissues to obtain enough GFP + cells. A future study should focus on GFP + cells solely in the Ctx and Cer, to elucidate the biology of *Slc16a2* expression in both brain areas in more detail and in a higher number of animals. Last, it would be of interest to study the role of MCT8 in TH transport and function during embryonal development. First pilot studies, using pregnant female MCT8-CreERT2 mice and one or three oral tamoxifen applications of 7.5 mg per mouse at diverse embryonal stages, have already shown promising but nonetheless still limited induction efficiencies, and are moreover hampered by the well-reported TAM-toxicity to pregnant females and their embryos (M. R. Sun et al. 2021)(Ved et al. 2019). Accordingly, while we demonstrate that MCT8-CreERT2 mice represent a suitable and versatile tool to study MCT8 expression and function following postnatal tamoxifen induction, a safe and effective protocol for embryonic induction remains to be established.

## Methods

### Animals

Animal experiments were conducted in compliance with European Union Directive 2010/63/EU and local regulations for the care and use of laboratory animals and approved by the animal ethics committee of the State of Bavaria, Germany; the study is reported in accordance with ARRIVE guidelines. SPF-mice were housed in IVC cages and maintained on a 12 h-dark–light cycle with free access to chow diet and water. Only male mice were assessed in this study, as MCT8-deficiency only affects male patients. Reporter lines were acquired from The Jackson Laboratory (Sun1-sfGFP: JAX #021039, Ai14: JAX #007914). Mct8-Cre and Mct8-CreERT2 mice were generated in house on a C57BL/6N background and are available upon request.

### Mouse line generation

Zygotes were injected with Cas9 protein, guide RNA targeting the *Slc16a2* start codon (Supplemental Table 2), and the DNA repair template with iCre-T2A or iCreERT2-T2A sequences. Micro-injected zygotes were transplanted into pseudo-pregnant females. Correct insertions were tested by PCR and Sanger sequencing in F0 offspring, and germline transmission confirmed in F1 mice (Supplemental File 2). Primers used for genotyping are indicated in Fig. 1a and listed in Supplemental Table 2, and the resulting bands are indicated in Fig. 1b.

### Tamoxifen application

Mice received 100 µL TAM in sunflower seed oil at 10 mg/mL per day, or oil as control, intraperitoneally (i.p.) for one, three, or five consecutive days, respectively. Mice that received TAM or VEH were housed in cages separated by treatment to prevent unintended induction. For oral gavage, mice received 200 µL of 10 mg/mL TAM in oil. All mice dosed with TAM or VEH received 5 mg/kg of the non-steroidal anti-inflammatory meloxicam (subcutaneously). Mice were euthanized for tissue processing one week after the first TAM administration.

### Tissues extraction

Mice were sacrificed by cervical dislocation, brains excised, and the MBH, 4 V ChP, Cer, LV ChP, and Ctx dissected and frozen in liquid nitrogen. For fluorescence imaging, mice were anesthetized using ketamine/xylazine and perfused through the heart with ice-cold PBS followed by 4% paraformaldehyde (PFA; 4% w/v, pH 7.4, Morphisto, 11,762.01000). Organs were postfixed in 4% PFA for 24 h and stored in PBS with 0.05% sodium azide at 4 °C. For vDISCO, animals were perfused with PBS containing heparin (25 U/ml, Ratiopharm, N68542.03) for 5–10 min and 4% PFA. After skinning, gut cleaning and rinsing in PBS, mice were post-fixed for 24 h in 4% PFA and stored in PBS with 0.05% sodium azide at 4 °C.

### Fluorescence-activated nuclei sorting (FANS)

MBH, Ctx with LV ChP, or Cer with 4 V ChP were transferred to a Dounce-homogenizer containing 700 µL (MBH) or 3 mL of ice-cold nuclei isolation buffer (25 mM sucrose, 25 mM KCl, 5 mM MgCl_2_, 20 mM Tris pH 8.0, 0.4% IGEPAL 630, 1 mM dithiothreitol (DTT), 0.15 mM spermine, 0.5 mM spermidine, 1 × phosphatase & protease inhibitor tablet, 0.4 units RNasin Plus RNase Inhibitor, 0.2 units SuperAsin RNase inhibitor) (Krishnaswami et al. 2016). Samples were homogenized by 10 pestle strokes with a looser pestle, 5 min incubation on ice, and 20 strokes with a tighter pestle. After filtering through a 20 µm cell strainer and pelleting by centrifugation at 1000 g for 10 min at 4 °C, 500 µL of staining buffer (RNAse-free PBS pH 7.4, 0.15 mM spermine, 0.5 mM spermidine, 0.4 units RNasin Plus RNase Inhibitor, 1.5% RNAse-free BSA, 1 µg/µL DAPI) were used for resuspension and samples were subjected to sorting on a FACS-Aria III (BD Biosciences) into 350 µL RLT buffer + DTT, then frozen.

### RNA extraction and qPCR

Tissues were homogenized in 500 µL (MBH, ChP) or 1 mL (Ctx, Cer) Quiazol using a Tissue Lyser II for 3 min at 30/sec. 100 µL or 200 µL chloroform were added after 5 min at RT, followed by shaking, 3 min of incubation and centrifugation at 12,000 × g for 15 min at 4 °C. RNA was isolated from supernatants using the RNeasy Micro Kit (QIAGEN GmbH 74004; MBH, ChP) or NucleoSpin RNA isolation kit (740,955, Machery-Nagel; Ctx, Cer) following the manufacturer’s instructions. Reverse transcriptions were performed using the QuantiTect® Reverse Transcription Kit (205,311, QIAGEN).

Sorted nuclei were mixed with 350 µL of 70% EtOH and transferred to columns of the RNeasy Micro Kit for RNA extraction, following the manufacturer’s instructions. cDNA was synthesized using the SMART-Seq V4 Ultra® Low Input RNA kit (634,888, Takara Bio; 11 cycles).

qPCRs were performed using the SYBR® Green PCR Master Mix (Applied Biosystems™) and specific primers (Supplemental Table 2) in a QuantStudio 7 Flex Real Time PCR System (Applied Biosystems™).

### Immunofluorescent staining

Perfused and post-fixed organs and brains were saturated with 30% sucrose in Tris-buffered saline (TBS, pH 7.2) for 48 h. Organs were sectioned into 16–20 μm slices using a cryostat and mounted on glass slides. 30 µm brain slices were stained free-floating. After washing with TBS, permeabilization and blocking was done using a solution of 0.25% (w/v) porcine gelatin and 0.5% (v/v) TritonX100 in TBS at RT for 1-2 h. Primary antibodies (Supplemental Table 3) were diluted in the same solution and incubated overnight at 4 °C. After washing with TBS, secondary antibodies (Supplemental Table 3) and DAPI were incubated for 2 h at RT. After washing and mounting, slides were sealed using Elvanol (150 mM Tris, 12% Mowiol 4–88, 2% DABCO) and imaged on a Leica SP5 confocal microscope or Zeiss Axio Scan 7 slidescanner.

### vDISCO

Perfused and fixed mice were placed in a glass chamber for washing, decolorization, decalcification, permeabilization, staining, and clearing according to the published methods (Cai et al. 2018, 2023; Pan et al. 2016). Sun1-sfGFP reporter detection was enhanced using the GFP-Booster Alexa-Fluor®-647 nanobody by ChromoTek (gb2AF647). Nuclei were stained with propidium iodide (PI; Sigma-Aldrich, P4864). LSFM images were acquired using an UltraMicroscope Blaze Imaging System and ImSpector imaging software from Miltenyi. Images were analyzed and processed using Imaris (Oxford Instruments) and syGlass Inc. virtual-reality software.

### Statistical analysis

Values in bar graphs are plotted as mean with SD if not stated differently. Statistical analysis was done using Graphpad Prism 8. T-tests were performed under the assumption of normal distribution, without assuming equal variance, and corrected for multiple comparisons using the Holm-Sidak method. No values were excluded.

Assessments were made unblinded in regards to genotype and treatment, but were reported in full faith.

## Supplementary Information

Below is the link to the electronic supplementary material.

Supplementary file1 (DOCX 48742 KB)

Supplementary file1 (video 12619 KB)

Supplementary file1 (video 12977 KB)

Supplementary file1 (video 13584 KB)

## Data Availability

The data collected from this study are available from the corresponding authors upon request.
