# Supplementary material for: A novel tamoxifen-inducible Mct8-CreERT2 mouse model for targeted studies of Mct8-expressing cells and thyroid hormone transport and function
Source: Transgenic Res. 2025 Nov 28;34(1):50. doi: 10.1007/s11248-025-00471-8 (PMC12660349; doi:10.1007/s11248-025-00471-8)
Supplement: Supplementary file 1 — Supplementary file1 (DOCX 48742 KB) [file 11248_2025_471_MOESM1_ESM.docx]

**A Novel Tamoxifen-Inducible Mct8-CreERT2 Mouse Model for Targeted Studies of Mct8-Expressing Cells and Thyroid Hormone Transport and Function**

Anna Molenaar, Noémi Mallet, Marin Bralo, Luciano J. Hoeher, Sonja C. Schriever, Ekta Pathak, Miriam Bernecker, Timo D. Müller, Ali Ertürk, Alberto Cebrian-Serrano, Paul T. Pfluger

**Supplemental Tables**

**Supplemental Table 1: Mct8 protein expression in rodent tissues by immunofluorescence (IF), immunohistochemistry (IHC), and Western Blotting (WB).** The table describes the tissues, the method and antibodies (Ab) used, the respective negative controls, the references and, if applicable, additional detection methods like qPCR or *in-situ* hybridization (ISH).

| Tissue | Method | Ab for IF, WB | Comment | Ref. |
| --- | --- | --- | --- | --- |
| Liver | IF, WB | Homemade C-terminal Rb-Ab | non-Mct8-injected oocytes provided as control | (Friesema et al., 2003) |
|  | WB | Homemade N-terminal Rb-Ab | WB of Mct8-KO tissues provided | (Wirth et al., 2009) |
| Heart | IF, WB | Homemade C-terminal Rb-Ab | non-Mct8-injected oocytes provided as control | (Friesema et al., 2003) |
| Kidney | IF, WB | Homemade C-terminal Rb-Ab | non-Mct8-injected oocytes provided as control | (Friesema et al., 2003) |
|  | IF, WB | C-terminal Rb-Ab by Eurogentec SA | no Mct8-KO control provided here (see below Ref(Trajkovic-Arsic et al., 2010)); not found on vendors website | (Becker et al., 2010) |
|  | IF, ISH | C-terminal Rb-Ab by Eurogentec SA | control in Mct8-KO (not shown), same Ab as above in Ref(Becker et al., 2010) (Lot differences cannot be excluded; here 1306) | (Trajkovic-Arsic et al., 2010) |
|  | WB | Homemade N-terminal Rb-Ab | WB of Mct8-KO tissues provided | (Wirth et al., 2009) |
| Brain | WB | Homemade C-terminal Rb-Ab | non-Mct8-injected oocytes provided as control | (Friesema et al., 2003) |
|  | IF, WB | Homemade N-terminal Rb-Ab | WB of MctT8-KO tissues provided | (Wirth et al., 2009) |
|  | IF | Commercial N-terminal (long isoform) Rb-Ab, HPA003353, Lot A61491  &  Commercial C-terminal Rb-Ab, NBP2-57308, Lot 100566 | Mct8-KO tissue provided as control; includes detailed supplementary table of Mct8 IF/IHC studies in human and rodent brains | (Wilpert et al., 2020) |
| Thyroid gland | IF, WB, qPCR | C-terminal Rb-Ab provided by Ian Simpson | Mct8-KO tissue provided as control | (Di Cosmo et al., 2010) |
|  | IHC | Commercial N-terminal (long isoform) Rb-Ab, HPA003353 | Mct8-KO provided as control; no Lot provided | (Wirth et al., 2011) |
|  | IF, WB, qPCR | Commercial N-terminal (long isoform) Rb-Ab by Sigma-Aldrich, HPA003353 | no Mct8-KO provided; no Lot provided | (Henning & Szafranski, 2016) |
| Retina | IHC, WB | IHC: Commercial Rb-Ab by MBL, BMP031  WB: Commercial C-terminal Rb-Ab by abcam, ab192828 | no Mct8-KO provided for IHC  WB of Mct8-KO tissues provided | (Bae et al., 2020) |
| Testes | IHC | Commercial Rb-Ab by MBL, BMP031 | no Mct8-KO provided for IHC | (Bae et al., 2020) |

**Supplemental Table 2: Guide RNA, genotyping primer, and qPCR primer sequences.**

| **Guide RNA sequence:** | |
| --- | --- |
| CGGGCTTGGCAGCGCCATCG | |
| **Genotyping primer sequences:** | |
| Extern For | CTTCGGGAGGCTGAAAGCCA |
| Extern Rev | AATTTGAAAAGGGAGTTTGGGGC |
| T2A Rev | GAGGGCAGAGGAAGTCTTCTAA |
| **qPCR primer sequences:** | |
| Mct8 For | CTCCTTCACCAGCTCCCTAAG |
| Mct8 Rev | ATGACGAGTGATGGTTGAAAGGC |
| Ttr For | TTGCCTCGCTGGACTGGTA |
| Ttr Rev | TTACAGCCACGTCTACAGCAG |
| Crym For | GGGAGTCATGCCTGCCTAC |
| Crym Rev | AGCCATTGCTGGGATCAAAGA |
| Malat1 For | GCAGTGTGCCAATGTTTCGT |
| Malat1 Rev | GCTGTTTCCTGCTCCGAGAT |

**Supplemental Table 3: Antibodies.**

| **Antibody** | **Species** | **Dilution** | **Ref and Lot** | **Vendor** |
| --- | --- | --- | --- | --- |
| Atlas Mct8 | Rabbit | 1:500 | Ref HPA003353  LOT 000024042 | Atlas Antibodies |
| Novus Mct8 | Rabbit | 1:500 | Ref NBP2-57308  LOT A117467 | Novus Biologicals |
| Alexa Flour 647 donkey anti rabbit IgG(H+L) | Donkey | 1:500 | Ref A31573  LOT 1903516 | Thermo Fisher Scientific |
| Alexa Flour 488 goat anti rabbit IgG(H+L) | Goat | 1:500 | Ref A11008  LOT 2304258 | Thermo Fisher Scientific |

**Supplemental Figures**

**
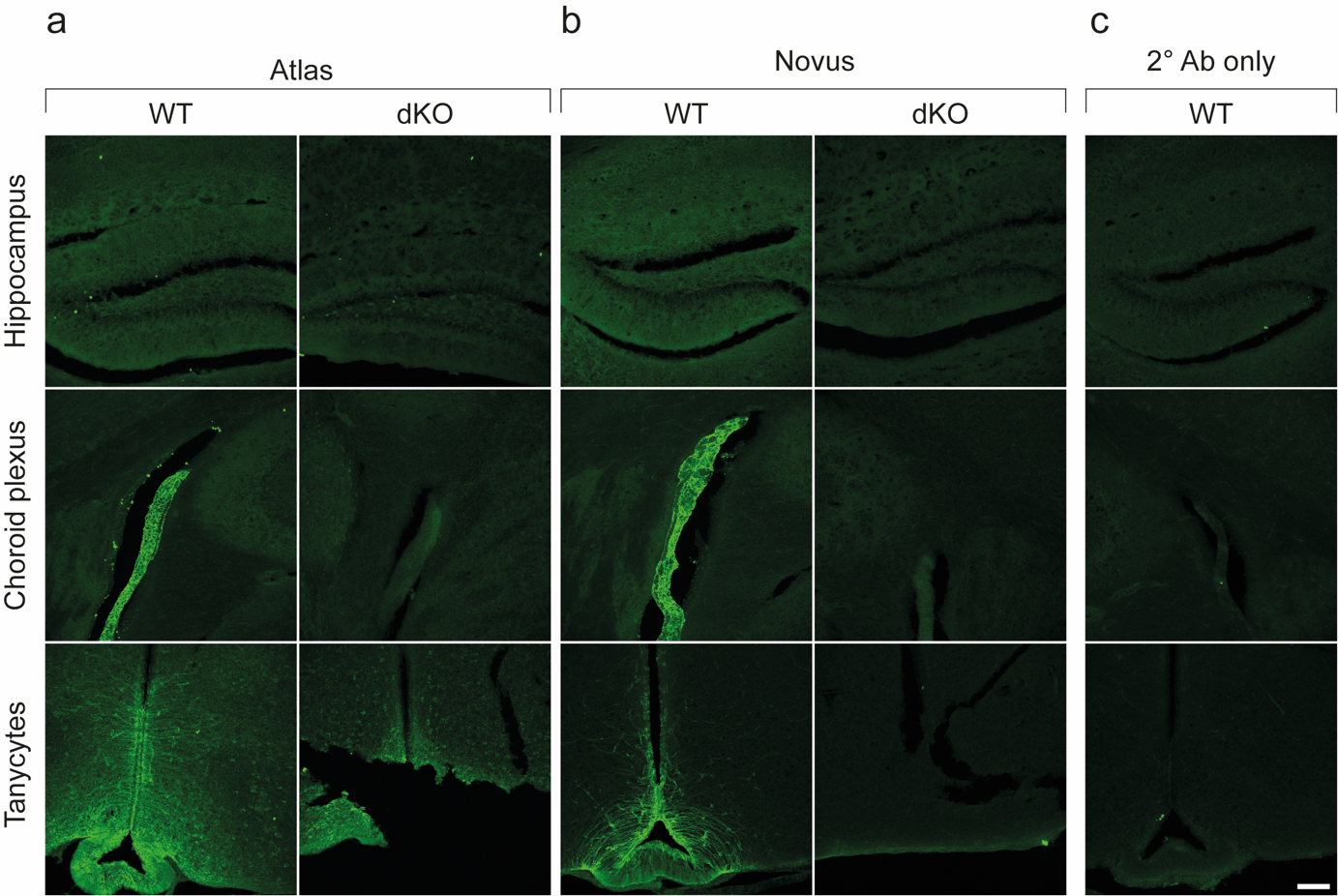
**

**Supplemental Figure 1: Performance of Atlas and Novus Mct8 antibodies in WT and double KO mouse brains.** To benchmark commercially available Mct8 antibodies, adult brain slices were stained (a) with Atlas antibody HPA003353 (lot 000024042) or (b) with Novus antibody NBP2-57308 (lot A117467) at a 1:500 dilution, comparing wild-type (WT) and Mct8/Oatp1c1 double KO (dKO) brains. (c) Brain slices were stained solely with the secondary antibody (2°Ab only; anti-Rb-488 1:500) to reveal unspecific staining. 20x magnification, scalebar = 100 µm.


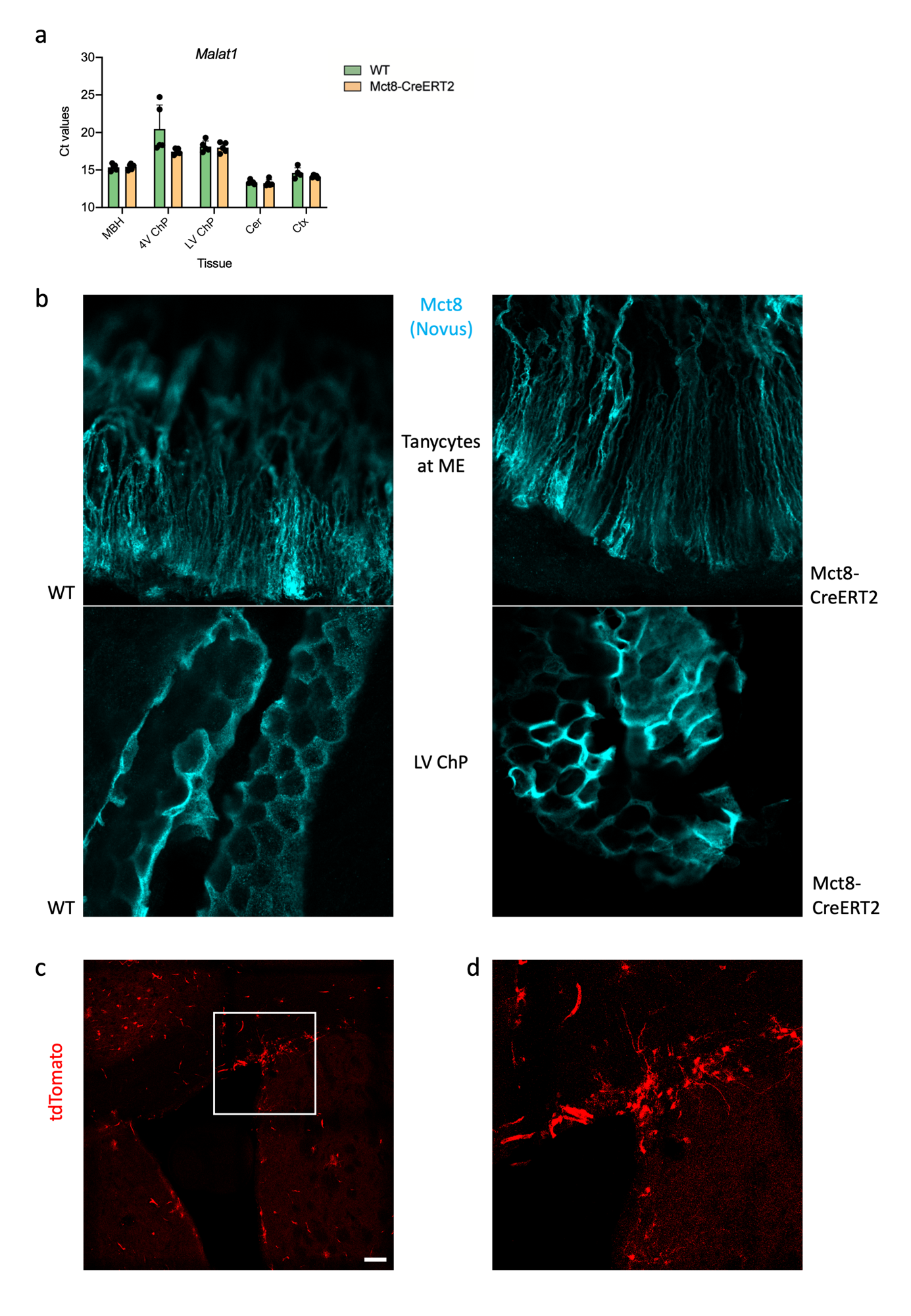


**Supplemental Figure 2: Additional data for the validation of the Mct8-CreERT2 line.** (a) Ct values of *Malat1* used in the qPCR of Fig. 1e showing no significant difference between WT and Mct8-CreERT2 knock-in. (b) Detailed localization of Mct8 in the tanycytes of the lower median eminence (ME) and the lateral ventricle (LV) ChP in WT and knock-in mice as shown by Mct8 antibody signal in 100x confocal microscopy images. (c) tdTomato pattern at the subventricular zone (SVZ) of an anterior brain slice of an Mct8-CreERT2;Ai14 mouse induced with 1 mg TAM i.p. for three days at 20x magnification (scalebar = 100 µm). (d) Magnification of box marked in (c).


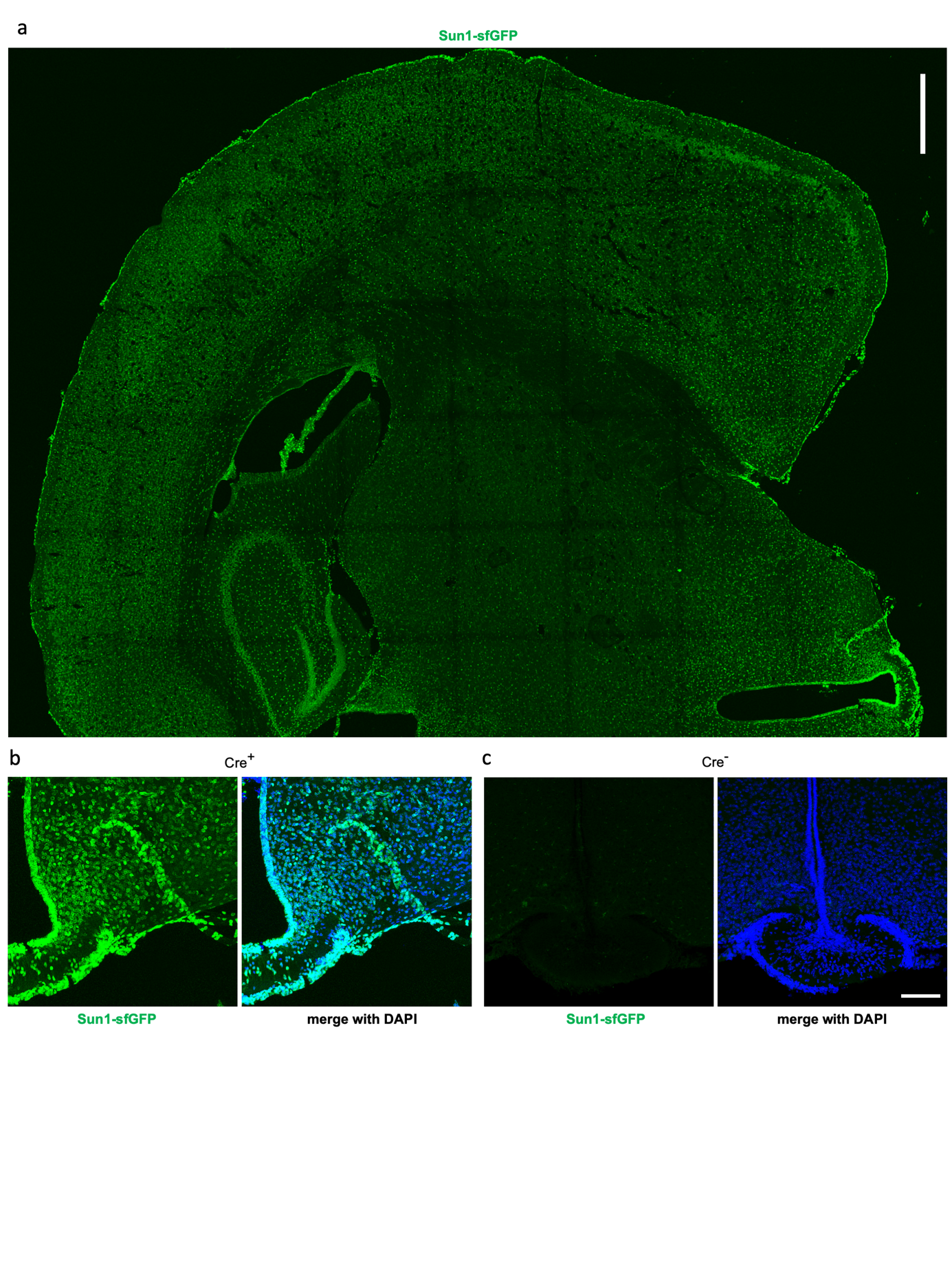


**Supplemental Figure 3: Ubiquitous Sun1-sfGFP expression in the non-inducible Mct8-Cre;Sun1-sfGFP mouse brain.** (a) Overview of Sun1-sfGFP signal in the whole brain slice. (b) Magnification of the MBH and overlap with DAPI signal reveals GFP signal around all nuclei. (c) Sun1-sfGFP signal is not present in Cre^-^ brains. (a) Scalebar = 500 µm. (b-c) Scalebar = 100 µm.


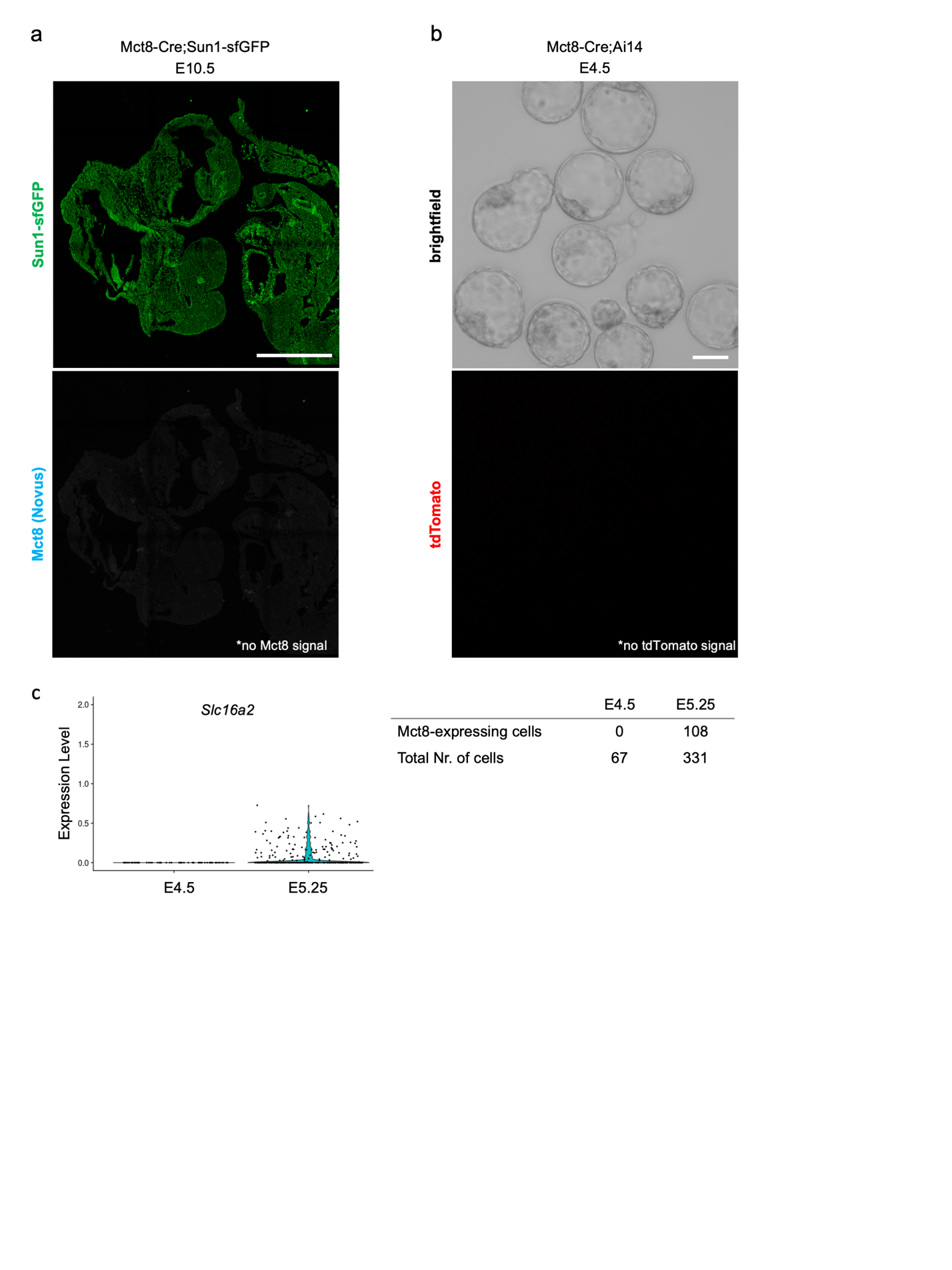


**Supplemental Figure 4: Mct8-Cre driven reporter activation in embryos vs blastocysts.** (a) Ubiquitous Sun1-sfGFP expression at E10.5 in MCT8-Cre;Sun1-sfGFP mice. Mct8 could not be detected by antibody staining at this stage. (b) Blastocysts (E4.5) of Mct8-Cre;Ai14 mice do not show reporter expression. (c) Analysis of publicly available RNA sequencing data confirms the absence of *Slc16a2* expression at day E4.5. At day E5.25 (implantation), expression was detected in one third of all analyzed cells (Mohammed et al. (Mohammed et al., 2017), and Cheng et al. (Cheng et al., 2019)). (a) Scalebar = 500 µm. (b) Scalebar = 2.5 µm.


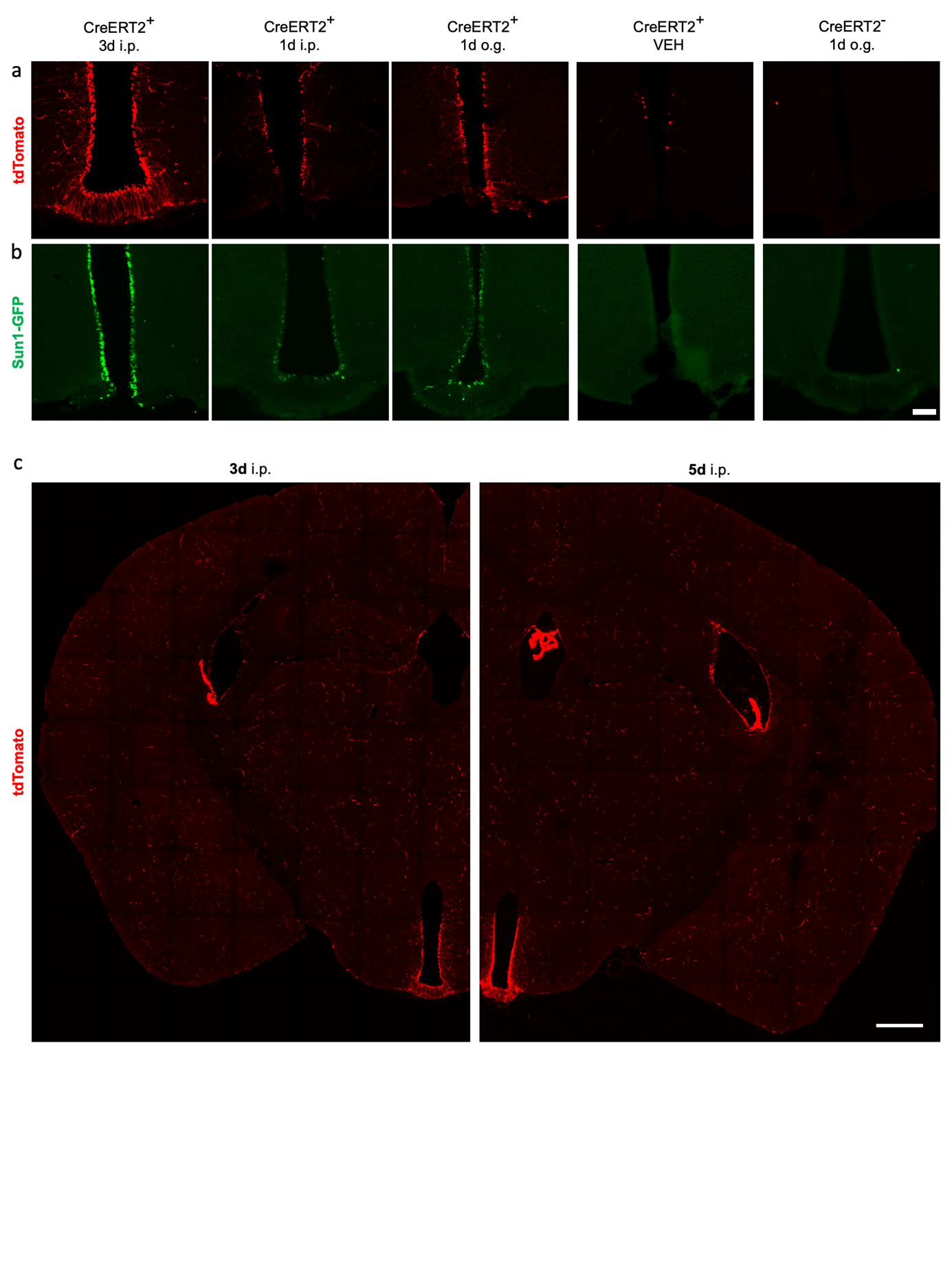


**Supplemental Figure 5: Induction efficiency and specificity of the Mct8-CreERT2 line.** Fluorescent reporter signal after 3d i.p., 1d i.p., or 1d o.g. administrations of 1 mg TAM per mouse <using (a) tdTomato and (b) Sun1-sfGFP reporter mice. Fluorescent reporter signal in uninduced Mct8-CreERT2;Ai14 and Mct8-CreERT2;Sun1-sfGFP mice injected with vehicle (VEH) is depicted as CreERT2+ VEH. Potentially leaky fluorescence reporter expression in Mct8-CreERT2-negative Sun1-sfGFP mice (CreERT2-) injected once via oral gavage with TAM (1d o.g.). (c) Comparison of 3d i.p. and 5d i.p induction of Mct8-CreERT2;Ai14 mice. (a-b) Scalebar = 100 µm. (c) Scalebar = 500 µm.


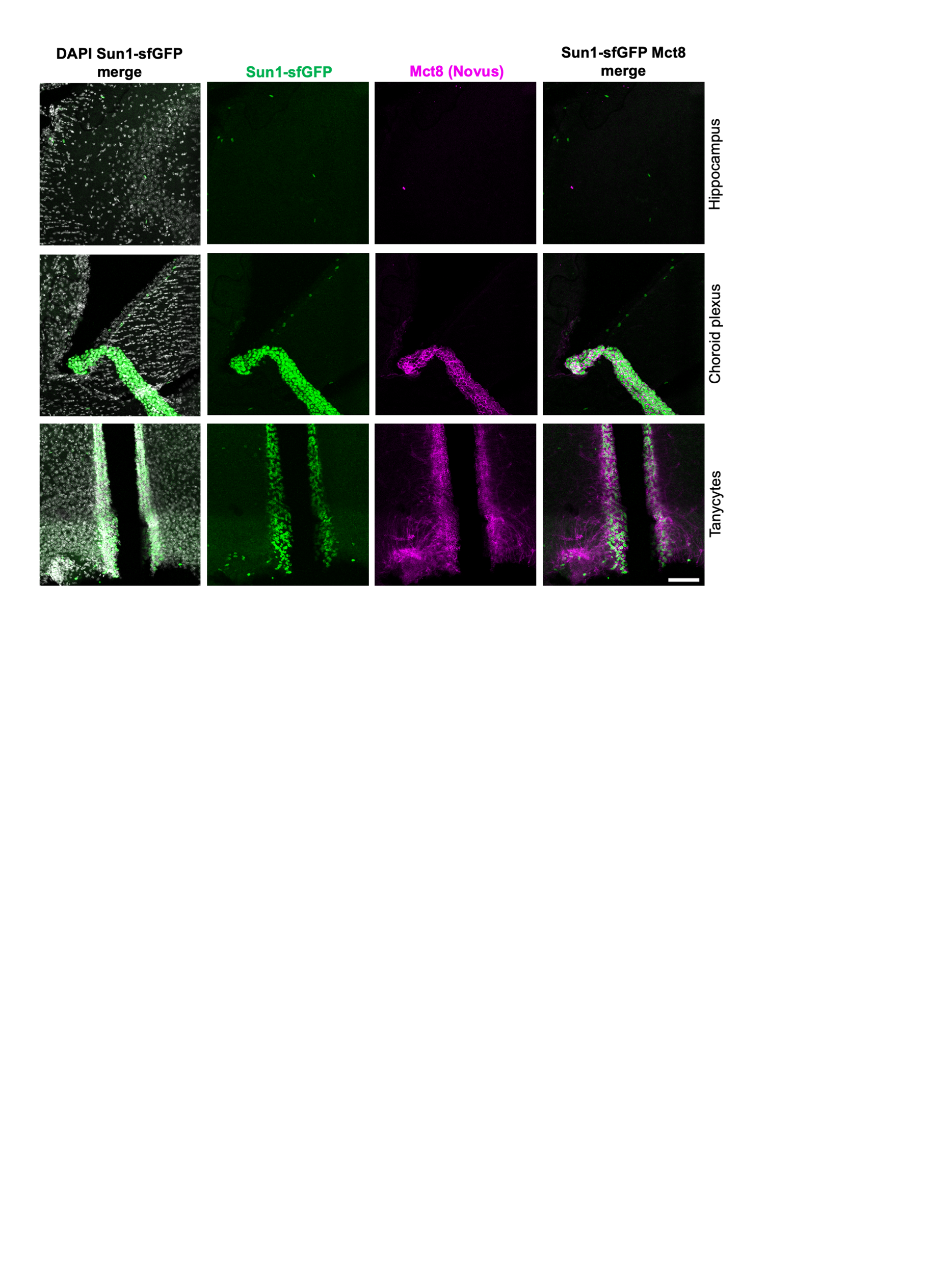


**Supplemental Figure 6: Sun1-sfGFP reporter signal and Mct8 antibody staining in Mct8-CreERT2;Sun1-sfGFP mouse brains.** Mct8-CreERT2;Sun1-sfGFP mice were induced three days with 1 mg TAM i.p. and sacrificed seven days later. Depicted are the hippocampus, ChP and MBH with tanycytes, including nuclear staining by DAPI, Sun1-sfGFP fluorescence enhanced with staining for GFP, Mct8 Ab-staining (Novus), and the respective merges. Scalebar = 100 µm.

**Supplemental Figure 7: Gating of Sun1-sfGFP^+^ nuclei from Mct8-CreERT2;Sun1-sfGFP brains during fluorescence-activated nuclei sorting (FANS).** Exemplary gating is shown for a Cer + 4V ChP sample, separating single nuclei from doublets and debris and then using the FITC-A channel to isolate Sun1-sfGFP^+^ nuclei.

**Supplemental References**

Bae, H. S., Jin, Y. K., Ham, S., Kim, H. K., Shin, H., Cho, G. bon, Lee, K. J., Lee, H., Kim, K. M., Koo, O. J., Jang, G., Lee, J. M., & Lee, J. Y. (2020). CRISPR/Cas9-mediated knockout of Mct8 reveals a functional involvement of Mct8 in testis and sperm development in a rat. *Scientific Reports 2020 10:1*, *10*(1), 1–9. https://doi.org/10.1038/s41598-020-67594-2

Becker, H. M., Mohebbi, N., Perna, A., Ganapathy, V., Capasso, G., & Wagner, C. A. (2010). Localization of members of MCT monocarboxylate transporter family Slc16 in the kidney and regulation during metabolic acidosis. *American Journal of Physiology - Renal Physiology*, *299*(1), 141–154. https://doi.org/10.1152/AJPRENAL.00488.2009

Cheng, S., Pei, Y., He, L., Peng, G., Reinius, B., Tam, P. P. L., Jing, N., & Deng, Q. (2019). Single-Cell RNA-Seq Reveals Cellular Heterogeneity of Pluripotency Transition and X Chromosome Dynamics during Early Mouse Development. *Cell Reports*, *26*(10), 2593-2607.e3. https://doi.org/10.1016/J.CELREP.2019.02.031

Di Cosmo, C., Liao, X. H., Dumitrescu, A. M., Philp, N. J., Weiss, R. E., & Refetoff, S. (2010). Mice deficient in MCT8 reveal a mechanism regulating thyroid hormone secretion. *The Journal of Clinical Investigation*, *120*(9), 3377–3388. https://doi.org/10.1172/JCI42113

Friesema, E. C. H., Ganguly, S., Abdalla, A., Manning Fox, J. E., Halestrap, A. P., & Visser, T. J. (2003). Identification of Monocarboxylate Transporter 8 as a Specific Thyroid Hormone Transporter. *Journal of Biological Chemistry*, *278*(41), 40128–40135. https://doi.org/10.1074/JBC.M300909200

Henning, Y., & Szafranski, K. (2016). Age-dependent changes of monocarboxylate transporter 8 availability in the postnatal Murine Retina. *Frontiers in Cellular Neuroscience*, *10*(AUG), 215097. https://doi.org/10.3389/FNCEL.2016.00205

Mohammed, H., Hernando-Herraez, I., Savino, A., Scialdone, A., Macaulay, I., Mulas, C., Chandra, T., Voet, T., Dean, W., Nichols, J., Marioni, J. C., & Reik, W. (2017). Single-Cell Landscape of Transcriptional Heterogeneity and Cell Fate Decisions during Mouse Early Gastrulation. *Cell Reports*, *20*(5), 1215–1228. https://doi.org/10.1016/J.CELREP.2017.07.009

Trajkovic-Arsic, M., Visser, T. J., Darras, V. M., Friesema, E. C. H., Schlott, B., Mittag, J., Bauer, K., & Heuer, H. (2010). Consequences of Monocarboxylate Transporter 8 Deficiency for Renal Transport and Metabolism of Thyroid Hormones in Mice. *Endocrinology*, *151*(2), 802–809. https://doi.org/10.1210/EN.2009-1053

Wilpert, N. M., Krueger, M., Opitz, R., Sebinger, D., Paisdzior, S., Mages, B., Schulz, A., Spranger, J., Wirth, E. K., Stachelscheid, H., Mergenthaler, P., Vajkoczy, P., Krude, H., Kühnen, P., Bechmann, I., & Biebermann, H. (2020). Spatiotemporal Changes of Cerebral Monocarboxylate Transporter 8 Expression. *Thyroid*, *30*(9), 1366–1383. https://doi.org/10.1089/THY.2019.0544

Wirth, E. K., Roth, S., Blechschmidt, C., Hölter, S. M., Becker, L., Racz, I., Zimmer, A., Klopstock, T., Gailus-Durner, V., Fuchs, H., Wurst, W., Naumann, T., Bräuer, A., De Angelis, M. H., Köhrle, J., Grüters, A., & Schweizer, U. (2009). Neuronal 3′,3,5-Triiodothyronine (T3) Uptake and Behavioral Phenotype of Mice Deficient in Mct8, the Neuronal T3 Transporter Mutated in Allan–Herndon–Dudley Syndrome. *Journal of Neuroscience*, *29*(30), 9439–9449. https://doi.org/10.1523/JNEUROSCI.6055-08.2009

Wirth, E. K., Sheu, S. Y., Chiu-Ugalde, J., Sapin, R., Klein, M. O., Mossbrugger, I., Quintanilla-Martinez, L., Hrabě De Angelis, M., Krude, H., Riebel, T., Rothe, K., Köhrle, J., Schmid, K. W., Schweizer, U., & Grüters, A. (2011). Monocarboxylate transporter 8 deficiency: altered thyroid morphology and persistent high triiodothyronine/thyroxine ratio after thyroidectomy. *European Journal of Endocrinology*, *165*(4), 555–561. https://doi.org/10.1530/EJE-11-0369
